# First Description of Non-Enzymatic Radical-Generating Mechanisms Adopted by *Fomitiporia mediterranea*: An Unexplored Pathway of the White Rot Agent of the Esca Complex of Diseases

**DOI:** 10.3390/jof9040498

**Published:** 2023-04-21

**Authors:** Samuele Moretti, Mary-Lorène Goddard, Alessandro Puca, Jacques Lalevée, Stefano Di Marco, Laura Mugnai, Eric Gelhaye, Barry Goodell, Christophe Bertsch, Sibylle Farine

**Affiliations:** 1 Laboratoire Vigne, Biotechnologies et Environnement UPR-3991, Université de Haute-Alsace, 33 rue de Herrlisheim, 68000 Colmar, France; mary-lorene.goddard@uha.fr (M.-L.G.);; 2 Laboratoire d’Innovation Moléculaire et Applications, Université de Haute-Alsace, Université de Strasbourg, CNRS, LIMA, UMR 7042, CEDEX, 68093 Mulhouse, France; 3 Department of Agricultural, Food, Environmental and Forestry Science and Technology (DAGRI), Plant Pathology and Entomology Section, University of Florence, P.le delle Cascine, 28, 50144 Firenze, Italy; laura.mugnai@unifi.it; 4 Institut de Science des Materiaux IS2M, Université de Haute-Alsace, CNRS, IS2M UMR 7361, F-68100 Mulhouse, France; 5 Institute of Bioeconomy, CNR, Via Gobetti, 101, 40129 Bologna, Italy; 6 Université de Lorraine, INRAE, IAM, F-54000 Nancy, France; 7 Department of Microbiology, University of Massachusetts, Amherst, MA 01003, USA; bgoodell@umass.edu

**Keywords:** *Fmed*, CMF, phenolates, ferric iron, ^•^OH, grapevine, GTDs

## Abstract

*Fomitiporia mediterranea* (*Fmed*) is the primary Basidiomycota species causing white rot in European vineyards affected by the Esca complex of diseases (ECD). In the last few years, an increasing number of studies have highlighted the importance of reconsidering the role of *Fmed* in ECD etiology, justifying an increase in research interest related to *Fmed*’s biomolecular pathogenetic mechanisms. In the context of the current re-evaluation of the binary distinction (brown vs. white rot) between biomolecular decay pathways induced by Basidiomycota species, our research aims to investigate the potential for non-enzymatic mechanisms adopted by *Fmed*, which is typically described as a white rot fungus. Our results demonstrate how, in liquid culture reproducing nutrient restriction conditions often found in wood, *Fmed* can produce low molecular weight compounds, the hallmark of the non-enzymatic “chelator-mediated Fenton” (CMF) reaction, originally described for brown rot fungi. CMF reactions can redox cycle with ferric iron, generating hydrogen peroxide and ferrous iron, necessary reactants leading to hydroxyl radical (^•^OH) production. These observations led to the conclusion that a non-enzymatic radical-generating CMF-like mechanism may be utilized by *Fmed*, potentially together with an enzymatic pool, to contribute to degrading wood constituents; moreover, indicating significant variability between strains.

## 1. Introduction

In Europe, grapevine trunk diseases (GTDs) are a major cause of vineyard decline: they have reached alarming levels of spread and incidence in several countries, with the Esca complex of diseases (ECD) being one of the most harmful and common throughout the Mediterranean region [1,2,3]. The ECD can considerably reduce the life of a vineyard, causing substantial economic losses in the order of millions of euros per year due to declines in yield and wine quality [4,5,6].

The ECD is considered to be a complex of multiple diseases, manifesting with internal damage to the wood and systemic damage to leaves and twigs [7]. Notwithstanding the role played by a broad mycoflora in ECD-affected grapevines, as evidenced by classical and omics approaches [8,9,10,11], the pathogens most frequently described in grapevine wood infections (for which Koch’s postulates have been at least partially fulfilled) are the two Ascomycota species *Phaeomoniella chlamydospora* [12] and *Phaeoacremonium minimum* [13], and usually prominent Basidiomycota species, which in Europe is most commonly *Fomitiporia mediterranea* (*Fmed*) [14,15]. The two Ascomycota species have been associated mostly with brown wood streaking, Petri disease, and grapevine leaf stripe disease (GLSD) [16,17], while *Fmed*, like other *Fomitiporia* spp. around the world, is a white rot agent associated with historical “Esca” (*sensu strictu*) and “Esca proper”; the latter being a condition where GLSD and white rot manifest simultaneously [7]. As the primary fungus responsible for white rot in grapevine woody tissue, *Fmed* is revealed to be the primary fungal agent behind the hydraulic, mechanical, and ecophysiological damage by the ECD in vines, stimulating considerable research interest related to vineyard ECD fundamental wood degradation mechanisms [18,19,20].

Basidiomycota species are the principal wood-decay taxa in nature, even though some Ascomycota and bacterial species are also able to induce or synergistically participate in some types of wood decay [21,22,23,24]. According to the dichotomous model of decay types historically proposed by Hartig [25] and still currently used, wood decaying Basidiomycota species can be broadly divided into white rot fungi (WRF) and brown rot fungi (BRF), based on the wood residue left being either bleached fibrous or brownish and cubic material, respectively [26]. In WRF, the breakdown of all wood components (lignin, hemicellulose, and cellulose) happens either progressively or simultaneously, depending on whether lignin is preferentially degraded with respect to holocellulose or not. More precisely, carbohydrate active enzymes (CAZymes) will be active on cellulose and hemicellulose (holocellulose), while laccases and class II peroxidases (Class II-PODs) will be active on lignin, and several auxiliary active (AA) redox enzymes will support cellulolytic and ligninolytic enzyme activity [27,28,29,30,31,32]. In contrast, in BRF, a reduced suite of CAZymes is typically active, while Class II-PODs are generally inactive or absent. The loss of POD function in the BRF has been documented via progressive genomic depletion as these fungi evolved from a white rot common ancestor [33]. The BRF attack and metabolize holocellulose primarily via non-enzymatic redox mechanisms, and they leave lignin repolymerized in a modified form after initial depolymerization and demethylation [29,34]. These redox mechanisms predominantly involve reactive oxygen species (ROS, mostly hydroxyl radicals) generated by a non-enzymatic pathway involving iron [35]. In the mid-1990′s, Goodell et al. introduced the “Chelator-Mediated Fenton (CMF) reaction” model for the brown rot agent *Gloeophyllum trabeum*, which was then further explored and refined in the following years for other Basidiomycota species [34,35,36,37,38,39,40] and recently also for some Ascomycota species [41,42].

The CMF reaction can be described as a mechanistic model in which a series of non-enzymatic chemical events takes place in the fungal extracellular matrix between the wood cell lumen and wood cell wall, involving solubilization of insoluble Fe(oxydydr)oxide complexes and reduction of iron. This ultimately leads to highly reactive oxygen species production, i.e., hydroxyl radicals (^•^OH), one of the strongest non-specific oxidants in biological systems [29,43]. The primary stages of the CMF model are (i) acidification of the wood cell lumen micro-environment and extracellular matrix surrounding fungal hyphae (down as low as pH 2) to facilitate iron solubilization and diffusion into the wood cell wall, mainly by secretion of small organic acids (mostly oxalic acid) capable of forming very stable Fe:organic acid complexes; (ii) production of low molecular weight compounds (LMWCs, mostly phenolates) that can bind and reduce iron and are able to diffuse into the wood cell wall through cell wall microvoids; (iii) reduction of iron in the higher pH environment of the wood cell wall (around pH 5.5), with LMWCs redox cycling with molecular oxygen, to yield Fe^2+^ and H_2_O_2_ respectively; and (iv) production of ^•^OH radicals via the Fenton reaction (Equation (1)) within the wood cell wall (and at a distance from the fungal hyphae to protect the fungus from oxidative damage). The highly reactive ^•^OH radical is then responsible for oxidative depolymerization of holocellulose and lignin, which can undergo further degradation via CAZymes and, if available, Class-II PODs [29,34,36,43,44,45].

(1) $${\mathrm{Fe}}^{2+}+H_{2}O_{2}\;\overset{\mathrm{pH}3-5.5}{\to }{\mathrm{Fe}}^{3+}+{\mathrm{OH}}^{-}+{\mathrm{OH}}^{•}$$

*Fomitiporia mediterranea* has historically been classified as a WRF [15]. In recent years, research has demonstrated how the binary classification of biomolecular decay mechanisms into either white rot or brown rot fungi is a “reductive monochromatic paradox” that does not represent all the nuances and peculiarities of Basidiomycota decay pathways [46,47,48]. In particular, as a WRF, *Fmed* presents a unique decay pattern fingerprint that results from a secretome that is unique in several different ways:

- CAZymes, laccases, class II-PODs (manganese peroxidases [MnPs] and generic peroxidases [GPs]), and other AAs have been reported in genomic, proteomic, and enzymological studies of *Fmed*, while genes encoding for versatile peroxidases (VPs) and lignin peroxidases (LiP) have been demonstrated to be absent in the *Fmed* genome [20,33,49,50,51];
- The *Fmed* genome possesses gene copy numbers with suggested involvement in LMWC biosynthesis, such as reducing polyketide synthases (*R-PKSs*), terpene synthases (*TSs*), and cytochrome P450 enzymes, proposed as important in LMWC-mediated brown rot decay mechanisms [33,47,52,53];
- LMWC production in vitro and the iron reduction capability of liquid culture supernatants have been previously reported for *Fmed*, but without separation of the high molecular weight fraction and without obtaining data on the redox cycling ability of the LMW metabolites [54,55,56,57];
- Even if described as a simultaneous white rot relying on in vitro wood mineralization assays, at the nanoscale, the wood cell wall decay pattern observed for *Fmed* does not resemble a typical enzymatic white rot decay pattern [20,51,58].

It has been conclusively demonstrated for both WRF and BRF that lignocellulolytic enzymes are too large to readily penetrate intact lignified secondary cell wall material. However, LMW metabolites from fungi are able to diffuse into the cell wall. This suggests that, in WRF, prior non-enzymatic LMWC-mediated degradation may be required or that the fungal extracellular enzymes remain confined to the fungal extracellular matrix [40,59,60,61,62,63]. Moreover, in vitro studies on the non-enzymatic degradation of different carbon sources or recalcitrant compounds used by selective WRF or LiP-deficient WRF showed that ferric (Fe^3+^) reducing-LMWCs were produced [64,65], efficiently oxidizing lignocellulose constituents in vitro when reacting with iron and H_2_O_2_ [38]. 

The peculiarities of the *Fmed*-decay pattern have demonstrated the lack of a validated biochemical mechanistic model for *Fmed*-induced white rot. Here we hypothesize that *Fmed* can trigger a non-enzymatic CMF-like pathway, potentially acting together with well-described enzymes. To address our hypothesis, we focused our research only on the low molecular weight *Fmed*-secretome, testing the fundamental steps of CMF chemistry, including: (i) the ability to produce ferric (Fe^3+^) reducing-LMWCs with ferric reducing activity (FeRA); and (ii) redox cycling capacity of the LMW fraction to generate H_2_O_2_ and ^•^OH radicals. We also tested for oxidative degradation in holocellulose and in the sawdust of Gewürztraminer grapevine wood (GW) when exposed to the LMW-*Fmed* extracts in order to verify in vitro the role of non-enzymatic mechanisms in wood constituent degradation. To include more variability, the research was conducted using different *Fmed* strains. A goal of this research is to help fill the knowledge gap on *Fmed* pathogenicity factors through the exploration of potential non-enzymatic mechanisms behind *Fmed*-induced white rot. This work may also help identify new paths for future research to potentially demystify ECD foliar symptom etiology in light of recent findings suggesting a role for white rot fungi in the development of ECD foliar symptoms in the northern hemisphere [19,66,67,68].

## 2. Materials and Methods

### 2.1. Fungal Strains and Culture Conditions

Six *Fomitiporia mediterranea* strains were used for these experiments, sourced from different mycological collections (Table S1). These strains have been previously used in other publications [20,23,51,69]. In this paper, an easy-to-read key legend has been assigned to each *Fmed* strain to avoid misinterpretation and facilitate the interpretation of the data presented. The strain *Fm1* was originally identified both morphologically and by sequencing of the Internal Transcribed Spacer (ITS) region in 2003 (NCBI GenBank accession number AY529683.1). Strains *Fm2*, *Fm3*, *Fm4*, *Fm5*, and *Fm6* were identified based on morphological aspects and population genetics studies [69]. In addition, strains *Fm2*, *Fm4*, *Fm5*, and *Fm6* were recently rapidly verified by molecular amplification of ITS sequences (% of similarity with *Fomitiporia mediterranea* in blast NCBI). The fungi were grown on 2% malt extract agar in darkness and kept at 27 °C until inoculation of the liquid culture.

### 2.2. Glassware Preparation

Because iron restriction conditions are a prerequisite for the production of ferric (Fe^3+^) reducing LMWCs, all glassware used throughout the experiments was carefully acid washed three times with 5.15 M nitric acid (HNO_3_) to remove any iron residue, then rinsed three times with Milli-Q^®^ water (18.2 MΩ·cm). 

### 2.3. Liquid Culture Experimental Design, Sampling, and Measurement of Fungal Growth

Liquid cultures were prepared using restricted nutrient and iron-free media to reproduce the nutrient restrictive conditions often found in wood and to stimulate LMW iron-binding compound production [70]. The cultures were prepared as follows: four Erlenmeyer flasks were inoculated for each *Fmed* strain listed in Supplementary Table S1. The liquid medium (200 mL per 500 mL Erlenmeyer flask) was prepared in Milli-Q^®^ water (18.2 MΩ·cm) according to Highley [71] with some modifications as follows: 0.25 g L^−1^ ammonium nitrate (NH₄NO₃; Duchefa Biochemie, Haarlem, The Netherlands), 2 g L^−1^ monobasic potassium phosphate (KH_2_PO_4_; Carl Roth GmbH, Karlsruhe, Germany), 0.5 g L^−1^ magnesium sulfate heptahydrate (MgSO_4_·7H_2_O; Carl Roth GmbH, Karlsruhe, Germany), 0.1 g L^−1^ calcium chloride (CaCl_2_; Carl Roth GmbH, Karlsruhe, Germany), 0.57 mg L^−1^ boric acid (H_3_BO_3_; VWR International LLC, Radnor, PA, USA), 0.31 mg L^−1^ zinc sulfate heptahydrate (ZnSO_4_·7H_2_O; Sigma-Aldrich, St. Louis, MO, USA), 0.039 mg L^−1^ copper sulfate pentahydrate (CuSO_4_·5H_2_O; VWR International LLC, Radnor, PA, USA), 0.036 mg L^−1^ manganese chloride tetrahydrate (MnCl_2_·4H_2_O; VWR International LLC, Radnor, PA, USA), 0.018 mg L^−1^ ammonium molybdate tetrahydrate ((NH_4_)_6_Mo_7_O_24_·4H_2_O; Carl Roth GmbH, Karlsruhe, Germany), and 0.001 g L^−1^ thiamine HCl (C_12_H_17_ClN_4_OS·HCl; Sigma-Aldrich, St. Louis, MO, USA). The carbon sources were 0.5 g L^−1^ glucose (Euromedex, Souffelweyersheim, France) and 50 µm microcrystalline cellulose (Acros Organics, Thermo Fisher Scientific Inc., Geel, Belgium) 1% (*w*/*v*). Iron was completely omitted. Liquid medium pH was adjusted before autoclaving to 5.5 using sodium hydroxide (NaOH) solution.

After autoclaving and cooling to room temperature, two 5 mm mycelial discs of *Fmed* were scraped from the surface of fully-grown petri dishes, avoiding agar contamination, and these were used for inoculation of the media in the Erlenmeyer flasks. Cultures were grown at 27 °C in darkness and under stationary conditions for a period of 12 weeks. Control flasks were prepared under the same conditions but uninoculated (mock). These negative controls were designated *C*_(-)_.

At the end of incubation, the total mycelium dry weight (DW) was determined by measuring the total dried solid mycelial mass following consecutive filtration steps. For this, an initial filtration using a 1 µm-pore glass fiber prefilter (Merck Millipore Ltd., Cork, Ireland) was carried out, followed by further steps of filtration using Whatman^TM^ cellulose membrane filters of progressively narrower pore size (0.45 µm, then 0.2 µm), and finally ultra-filtration with Polyethersulfone (PES) 5 Kda filters (Biomax^®^, EMD Millipore Corporation, Billerica, MA, USA). To facilitate the process, a vacuum pump (Gardner Denver Welch Vacuum Technology Inc., Monroe, LA, USA) coupled with Nalgene^TM^ filter holders and receivers (Nalgene Nunc International Corporation, Rochester, NY, USA) was used. The filtrate was lyophilized to reduce the liquid volume to approximately one-third and stored at −80 °C until extraction of LMWCs. Filters containing the mycelium were dried at 60 °C to obtain the dried mycelial biomass weight. 

### 2.4. LMWC Extraction

In order to extract the phenolic and phenolic-derived LMWCs, the medium was acidified (pH 3) with 1N HCl, and a triple ethyl acetate extraction (1:1 volume ratio) was conducted [70], followed by rotary evaporation under reduced pressure (Laborota 4000, Heidolph Instruments GmbH & Co., KG, Schwabach, Germany). The residual LMW fractions were resuspended in methanol (MeOH, LC-MS quality), filtered through a 0.2 µm syringe filter, then stored at −20 °C for further analysis.

### 2.5. Determination of Total Phenols

Total phenols in the LMW methanolic extract were determined according to the method described by Perez-Gonzalez et al. [41] with some modifications. Briefly, samples of extract (40 μL) and the Folin–Ciocalteu reagent (200 μL) were reacted (5 min at room temperature) before the addition of 5% sodium carbonate (600 μL) to initiate the reaction. Initial sodium carbonate concentration was reduced, as in the process employed by Cicco and Lattanzio [72], to avoid precipitation. After a further reaction period (90 m at room temperature) in the dark, absorbance values at 765 nm were determined spectrophotometrically (Agilent Cary 8454 UV-Visible, Agilent Technologies Australia Pty Ltd., Mulgrave, Victoria, Australia) [73], with gallic acid solutions used as phenolic concentration standards. Methanolic extract fractions were replaced with MeOH-only for blanks, while negative controls (*C*_(-)_) contained an aliquot of methanolic extract of the liquid mock-inoculated medium (to check background noise).

### 2.6. LMW Fraction Analysis

LMW methanolic extracts (49 µL), with 1 µL of a 1 mg/mL methanolic solution of 5-methylsalicylic acid added as an internal standard, were used for HPLC analysis. Ultra-high-performance liquid chromatography (UHPLC) coupled to an Impact II Q-TOF-MS/MS mass spectrometer equipped with an electrospray ESI source Apollo II operating in negative mode (Bruker Daltonics GmbH, Bremen, Germany) was used with a reverse-phase C18 Bruker Intensity Solo HPLC column (2.0 μm particle size, 100 Å pore size, and dimensions of 2.0 mm × 100 mm) (BRHSC18022100, Bruker Daltonics GmbH, Bremen, Germany). The mobile phases were ultrapure water with 0.1% of formic acid (eluent A) and MeOH with 0.1% of formic acid (eluent B) and were used in gradient expressed as a percentage of B: 2 min at 1.0%, linear gradient during 15 min until 99%, 3 more min at 99%, followed by 5 min in the initial conditions (1% B) for equilibration before a new injection. The total flow rate was 0.25 mL/min, the injection volume was 10 µL, and the oven column and autosampler temperatures were 35 and 8 °C, respectively. The MS detector was internally calibrated before starting the batch analysis, and additionally, at the beginning of each injection, spiked with 10 mM sodium formate solution in isopropyl alcohol:H_2_O (1:1, *v*/*v*). The ESI parameters were as follows: capillary voltage −3500 V, end plate offset −500 V, nebulizer gas 29.0 psi, dry gas 8.0 L/min, and dry temperature 200 °C. The spectral rate was 8 Hz over a 20–1600 m/z mass range in auto MS/MS scan mode. A collision energy ramp was applied from 20 to 50 eV. Data acquisition was achieved by Bruker Compass HyStar 5.1, and otofControl 5.2 software and data treatment was performed with Data Analysis 5.3 and METABOSCAPE 2021b (Bruker Daltonics GmbH, Bremen, Germany). Relative counts (Rc) of specific LMWC structures were used to solely compare quantities of individual compounds among *Fmed* strains.

### 2.7. Determination of Ferric Iron Reduction Activity (FeRA) in the LMW Fraction

LMW methanolic extracts were tested for ferric iron reduction activity (FeRA), following the protocol of Perez-Gonzalez et al. [41]. A reaction mix at the following final concentration was prepared: 100 µM acetate buffer (pH 5.5), 250 µM of freshly prepared aqueous FerroZine^TM^ (Acros Organics, Thermo Fisher Scientific Inc., Geel, Belgium), and 300 µM of freshly prepared FeCl_3_. LMW methanolic extracts, with their LMW total phenol concentration adjusted to 150 µM according to Folin–Ciocalteu results. They were used to verify if the differences in iron reduction between strains were due to respective total phenol concentrations or different ratios among phenol types. Blank and control solutions were prepared as in Section 2.5.

All procedures were conducted in the absence of light and at room temperature. Fe^3+^ reduction was assayed spectrophotometrically at 562 nm (Agilent Cary 8454 UV-Visible, Agilent Technologies Australia Pty Ltd., Mulgrave, Victoria, Australia), monitoring the absorbance after 45 min of incubation with the ferrozine reaction mixture [74], using FeCl_2_ solutions as standards.

### 2.8. Determination of Hydrogen Peroxide Levels by Ferrous Oxidation in the Presence of Xylenol Orange (FOX Assay) 

LMW methanolic extracts were tested for hydrogen peroxide generation during the oxidation of ferrous iron in the Fenton reaction using the ferrous ammonium–xylenol orange (FOX) assay and the method proposed by Perez-Gonzalez et al. [41], with a few modifications. A stock FOX solution containing Pierce^TM^ Peroxide Assay Reagent B (Pierce Biotechnology, Thermo Fisher Scientific Inc., Rockford, IL, USA) was prepared by dissolving ferrous ammonium sulfate directly in H_2_SO_4_ to avoid the spontaneous oxidation of ferrous ions at neutral pH. A LMW methanolic extract (buffered in 50 mM MES, pH 5.5) was then mixed with the FOX reagent to yield a final solution of 150 µM phenolics (as determined by the Folin-Ciocalteu assay), 100 µM of xylenol orange, 250 µM of ferrous ammonium sulfate, and 25 mM of H_2_SO_4_. Blank and control solutions were prepared as in Section 2.5 and Section 2.7.

The reaction mix was incubated for 30 min in the dark before spectrophotometric determination (Agilent Cary 8454 UV-Visible, Agilent Technologies Australia Pty Ltd., Mulgrave, Victoria, Australia) at 560 nm [75]. H_2_O_2_ was used for calibration solutions.

### 2.9. Determination of Hydroxyl Radical Activity by Electro Paramagnetic Resonance (EPR)

LMW methanolic extracts from the different *Fmed* strains were evaporated to dryness (45 min at 35 °C) using an Eppendorf Concentrator (Eppendorf AG, Hamburg, Germany), then resuspended either in sodium acetate buffer at pH 5.5 or acidified down to pH 3.5, to check the capacity of the LMWCs to generate hydroxyl radicals both at the pH of the intact wood cell wall (5.5) and in the more acidic environment during decay (3.5). For ^•^OH detection, 5,5-Dimethyl-1-pyrroline N-oxide (DMPO, TCI Europe N.V., Zwijndrecht, Belgium) was used as a spin-trap agent in EPR. The final reaction mixture for each sample was prepared according to the process used by Perez-Gonzalez et al. [41] with a few modifications by adding (in the following order): an aliquot of LMW methanolic extract, H_2_O_2_ (0.15 mM), DMPO (100 mM), and finally Fe^3+^ (0.15 mM) to start the reaction. An aliquot of the methanolic extract from the uninoculated liquid medium was used as a control to check the background Fenton reaction. After mixing and incubation at room temperature for 5 min (in the absence of light), the mixture was transferred to a 50 µL capillary glass tube for EPR analysis. An X-Band ESR spectrometer (Magnettech MS400) was used for these analyses. 

The DMPO-OH spectrum was simulated by PEST WinSIM software [76] in order to check the identity of the radical adduct. The simulation parameters were 100% Gaussian line shape, 1.16-G linewidth, and hyperfine coupling constants *a*H = 14.85 and *a*N = 14.96 G. Semi-quantification of ^•^OH relative amplitudes were represented by plotting the average amplitude of the tallest peak of DMPO-OH adduct spectra for all *Fmed* strain LMW fractions.

### 2.10. Carbohydrate Oxidative Degradation Assays

To evaluate the oxidative effect of any CMF reactions promoted by Fe^3+^-reducing LMWCs on holocellulose, microcrystalline cellulose and hemicellulose (xylan) sources were used, following the process described by Arantes and Milagres [77]. Grapevine wood sawdust produced from debarked clear wood (free of visible defects) from Gewürztraminer vines (GW-sawdust) was used to evaluate oxidation directly on the wood material. Following procedures from Arantes et al. [38] with some modifications, a mixture of 1% (*w*/*v*) microcrystalline cellulose, xylan, or GW-sawdust was mixed with 50 mM sodium acetate buffer (pH 5.5), 100 µL of LMW *Fmed* methanolic extract, 40 mM H_2_O_2_, and freshly prepared 4 mM FeCl_3_; the last of which was added at the end to start the reaction. The mixture was incubated at 50 °C for 12 h (hrs). After incubation, 1.5 mL of 1% (*w*/*v*) 3, 5 dinitro salicylic acid (DNS) reagent was added to each sample, and the mixture was boiled for 5 min and cooled in ice to stop the reaction. For each reaction, a pool of biological replicates was used. Absorbance at 540 nm was measured to assess the release of reducing sugars in the carbohydrates. A control with methanolic extract using a liquid medium mock-inoculated (*Fmed* LMW-Fe^3+^-reductants free) was used to check holocellulose oxidation only in the presence of Fe^3+^ and H_2_O_2_.

### 2.11. Statistical Analysis 

The experiments were set up in a randomized design. Data distribution was checked using D’Agostino and Pearson and Kolmogorov–Smirnov’s tests, and homoscedasticity for normally distributed data was verified using Bartlett’s test. To highlight differences between *Fmed* strains, data from DW (g) biomass (*n* = 3) and total phenols (*n* = 3) were analyzed by one-way ANOVA, and groups of means were separated by Fisher’s LSD multiple comparison tests (*p* < 0.05). Data from pH in the liquid medium (*n* = 3) were analyzed by Welch’s ANOVA. Data from LMWCs Rc (*n* = 4), µmol L^−1^ of Fe^2+^ reduced from Fe^3+^ and µmol L^−1^ of generated H_2_O_2_ (*n* = 3), the relative amplitude of ^•^OH-adducts in EPR at pH 3.5 (*n* = 4) were assessed using a Kruskal–Wallis test, and mean ranks were separated according to Dunn’s multiple comparison tests (*p* < 0.05). The relative amplitude of ^•^OH-adducts in EPR at pH 5.5 (*n* = 3) was analyzed using the unpaired two-tailed *t*-test (*p* < 0.05). Graphs and statistical elaboration were made using Prism8 (GraphPad Software, San Diego, CA, USA).

## 3. Results

### 3.1. Fungal Biomass

**Figure 1:**
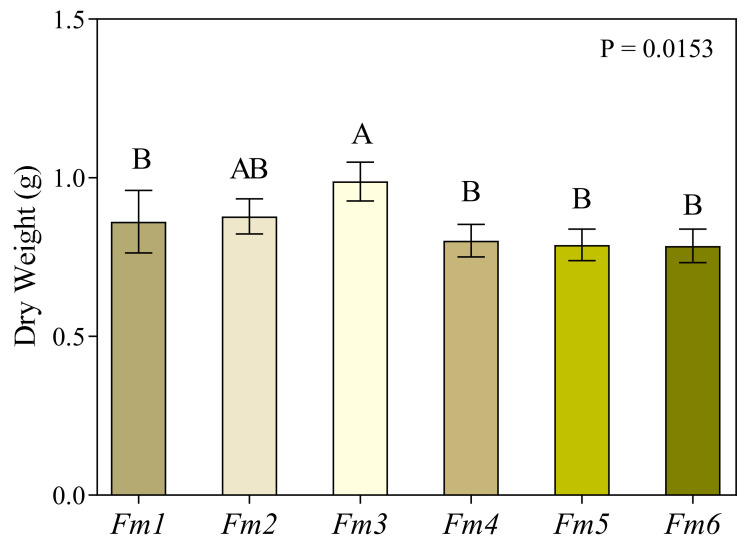
Dry weight of filtered liquid culture from 6 strains of *Fmed* (*Fm1*, *Fm2*, *Fm3*, *Fm4*, *Fm5*, and *Fm6*) grown at 27 °C under static conditions for 12 weeks in the dark. Values are the means ± standard deviation (*n* = 3). Results were analyzed by one-way ANOVA, followed by Fisher’s LSD multiple comparison test (*p* < 0.05). Different letters indicate significant differences according to multiple comparison test results.

Fungal biomass production measured at the end of the experiment showed that strain *Fm3* produced the highest quantity of dry biomass, while *Fm5* and *Fm6* were the strains that produced the least (−20.56% and −20.23% compared to *Fm3*, respectively). The other strains produced a statistically comparable amount of biomass (Figure 1). 

### 3.2. Culture Media Acidification

**Figure 2:**
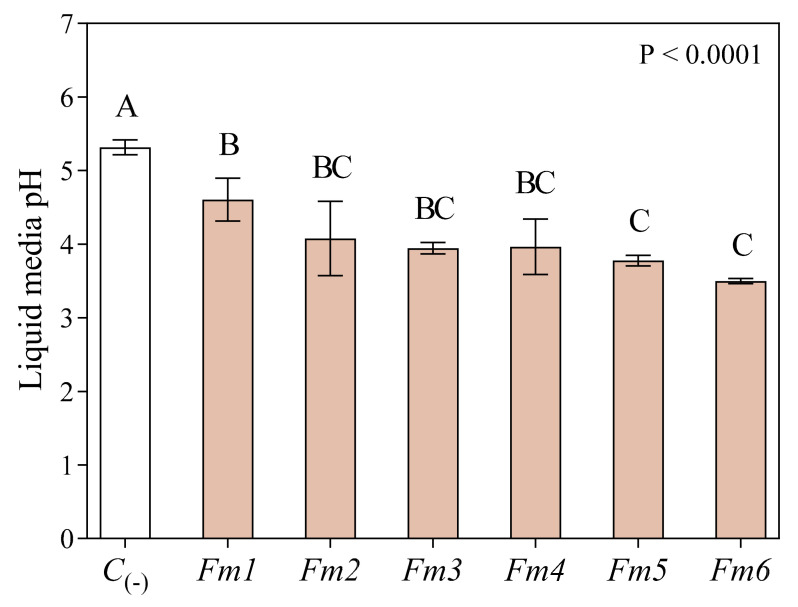
Liquid media pH at the end of the experiment for the 6 strains of *Fmed* (*Fm1*, *Fm2*, *Fm3*, *Fm4*, *Fm5*, and *Fm6*) and in control (*C*_(-)_)flasks. Values are the means ± standard deviation (*n* = 3). Results were analyzed by Welch’s ANOVA (*p* < 0.05). Different letters indicate significant differences according to multiple comparison test results.

Culture media pH was measured at the end of the experiment. General acidification with respect to initial pH (5.5) was observed in all liquid cultures, with the lowest pH value recorded for the strains *Fm5* (3.8) and *Fm6* (3.5) (Figure 2). The exception was the negative control (*C*_(-)_), where the pH of the liquid media remained almost the same (5.3) from the beginning of the experiment. Liquid media from strain *Fm1* culture had the highest pH values (4.6, +21.06% compared to *Fm5*, and +31.43% compared to *Fm6*). *Fm6* was responsible for the greatest amount of liquid media acidification relative to the initial pH, while *Fm1* showed the lowest media acidification.

### 3.3. Total Phenols Analysis

**Figure 3:**
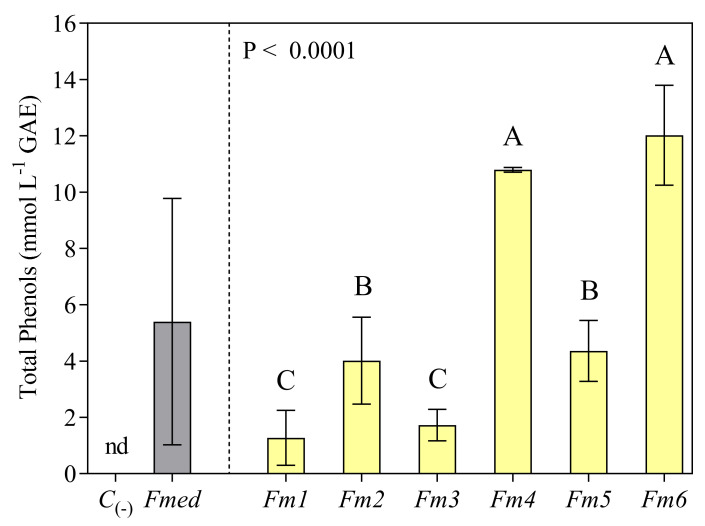
Total phenols from LMW methanolic extracts analyzed spectrophotometrically using a Folin–Ciocalteu assay. Values (expressed as mmol L^−1^ gallic acid, GAE) are the means ± standard deviation (*n* = 3). Results from the negative control (*C*_(-)_) and *Fmed* average value are shown on the left (nd = not detected). Results from different *Fmed* strains (*Fm1*, *Fm2*, *Fm3*, *Fm4*, *Fm5*, and *Fm6*) were analyzed by one-way ANOVA, followed by Fisher’s LSD multiple comparison test (*p* < 0.05). Different letters indicate significant differences according to multiple comparison test results.

Total phenols were measured in the methanolic LMW extracts, with an average value for *Fmed* of 5.6 mmol L^−1^ (Figure 3). The highest values were recorded in strain *Fm6* (12.02 mmol L^−1^). The strain with the lowest values (1.27 mmol L^−1^) was *Fm1* (−89.43% compared to *Fm6*). The other strains produced lower levels of phenols with respect to *Fm6* in the following order: *Fm6* > *Fm4* > *Fm5* > *Fm2* > *Fm3* > *Fm1* (Figure 2).

### 3.4. FeRA and Hydrogen Peroxide Generation from LMWC Redox Cycle

**Figure 4:**
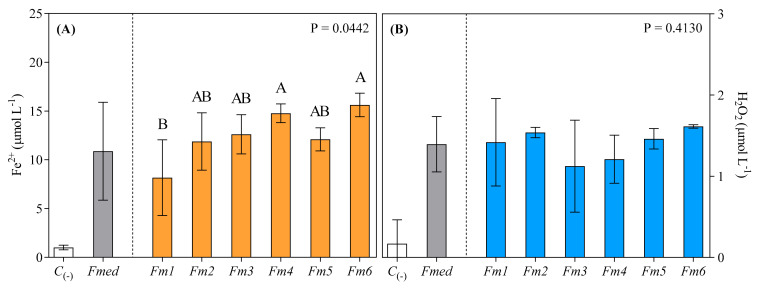
Ferrous iron (expressed as µmol L^−1^ of Fe^2+^) reduced from ferric iron (**A**) via the fungal secreted LMWCs and H_2_O_2_ (expressed as µmol L^−1^) generated by redox cycling of LMWCs with Fe^2+^ and O_2_ (**B**). Values are the means ± standard deviation (*n* = 3). Results from negative controls (*C*_(-)_) and *Fmed* average values are shown on the left of each graph. Data from different *Fmed* strains (*Fm1*, *Fm2*, *Fm3*, *Fm4*, *Fm5*, and *Fm6*) were analyzed by the Kruskal–Wallis test, followed by Dunn’s multiple comparison tests (*p* < 0.05). Different letters indicate significant differences according to multiple comparison test results.

FeRA and hydrogen peroxide generated from redox cycling of the methanolic fungal LMW extracts were determined for the different strains of *Fmed*, with all extracts found to reduce ferric to ferrous iron (Figure 4A: average Fe^2+^ value for *Fmed* of 12.52 µmol L^−1^). All extracts were also found to generate hydrogen peroxide from LMWCs redox cycling (Figure 4B: average H_2_O_2_ value of 1.4 µmol L^−1^). Strains *Fm6* (15.62 µmol L^−1^) and *Fm4* (14.76 µmol L^−1^) reduced the most iron, while strain *Fm1* reduced the least (8.16 µmol L^−1^, −47.76%, and −44.72%, compared to *Fm6* and *Fm4*, respectively). All extracts from the other *Fmed* strains reduced statistically comparable amounts of ferric iron, but all reduced significantly less iron compared to *Fm6* (Figure 4A). LMWCs redox cycling of extracts from different *Fmed* strains generated statistically comparable amounts of H_2_O_2_, even though the highest absolute values were recorded for strains *Fm1* and *Fm6* (1.42 and 1.61 µmol L^−1^, respectively) and the lowest for strain *Fm3* (1.12 µmol L^−1^, −21.13% compared to *Fm1* and −30.43% compared to *Fm6*) (Figure 4B).

### 3.5. LMWC Fraction Characterization

**Figure 5:**
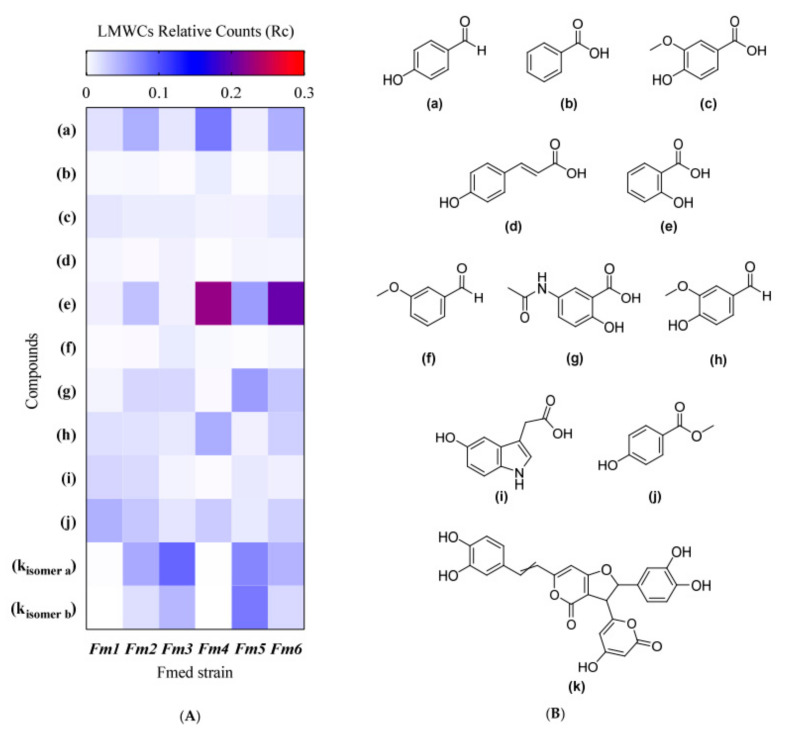
Heatmap (**A**) shows the LMW compounds’ average relative counts (Rc) analyzed by UHPLC-MS/MS. Chemical structure (**B**) of described compounds: (**a**) 4-hydroxybenzaldehyde, (**b**) benzoic acid, (**c**) 4-hydroxy-3-methoxybenzoic acid (vanillic acid), (**d**) 4-hydroxycinnamic acid (*p*-coumaric acid), (**e**) 2-hydroxybenzoic acid (salicylic acid), (**f**) 3-methoxybenzaldehyde, (**g**) N-acetyl-5-aminosalicylic acid, (**h**) 4-hydroxy-3-methoxybenzaldehyde (vanillin), (**i**) 5-hydroxyindole-3-acetic acid, (**j**) methyl-4-hydroxybenzoate, (**k**) hypholomine B (isomers a and b). Values of Rc in the heatmap are the means of four replicates (*n* = 4). Statistical analysis results for each compound are shown in Table S2.

**Figure A1:**
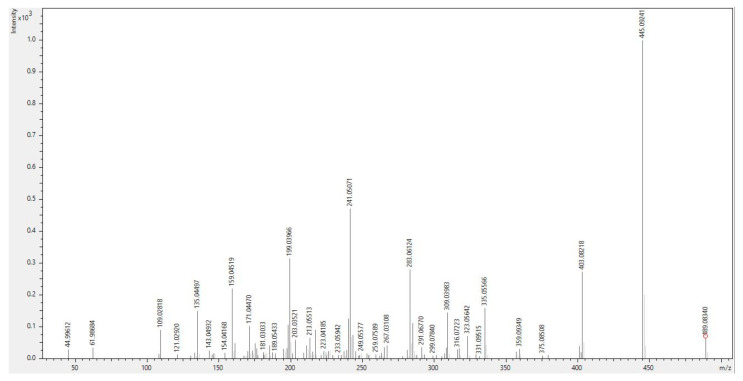
MS/MS spectrum of compound (k) annotated as hypholomine B.

Several phenolic acids (hydroxybenzoic and hydroxycinnamic acids), phenolic aldehydes, and other aromatic compounds were identified from the LMW methanolic extracts of all *Fmed* strains (Figure 5, Table 1; Appendix A, Figure A1, Table A1 and Table A2). Some of the identified LMWCs have previously been reported to chelate and reduce ferric iron (see list of compounds in Table 1). For 3-methoxybenzaldehyde (f), N- acetyl-5-aminosalicylic acid (g), 5-hydroxyindole-3-acetic acid (i), methyl-4-hydroxybenzoate (j), and the phenolic pigment hypholomine B (k), further analyses are necessary to determine their FeRA. No strain-specific compounds were identified. Because standards could not be obtained for most compounds, the Rc of specific LMWC structures can be compared across all samples, but comparative quantification between different compounds in the same sample cannot be performed. Except for hypholomine B (not detected in the extracts of the strains *Fm1* and *Fm4*), all the identified compounds were produced by all strains, although in different amounts. Strain *Fm1* produced the most methyl-4-hydroxybenzoate, with strain *Fm3* producing the most hypholomine B (isomer a), and strain *Fm5* producing the most hypholomine B (isomer b). Strain *Fm4* produced the most 4-hydroxybenzaldehyde and salicylic acid (Figure 5 and Table S2). 

### 3.6. Determination of Hydroxyl Radical Activity by Electron Paramagnetic Resonance (EPR)

**Figure 6:**
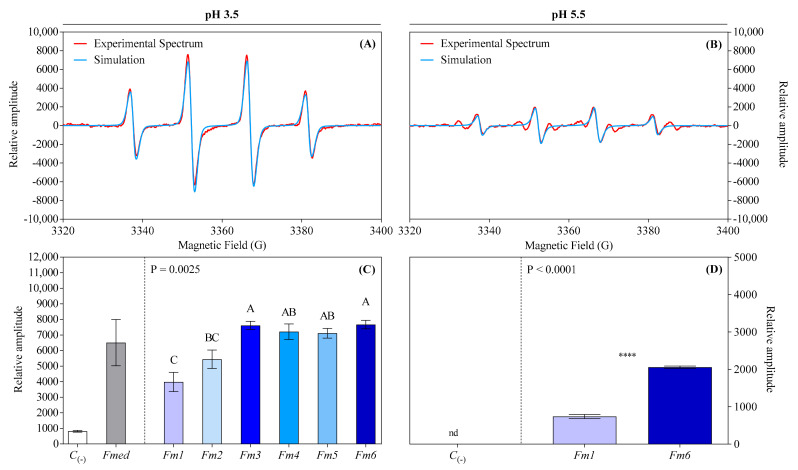
Hydroxyl radical sample spectrum compared with theoretical simulation (**A**,**B**) and relative amplitude (**C**,**D**) generated from LMWCs redox cycle with iron, analyzed using spin-trapping in EPR. Values are the means ± standard deviation: for the reaction at pH 3.5 (**C**; *n* = 4), results from negative controls (*C*_(-)_) and *Fmed* average values are shown on the left. Results from different *Fmed* strains (*Fm1*, *Fm2*, *Fm3*, *Fm4*, *Fm5*, and *Fm6*) were analyzed using the Kruskal–Wallis test, followed by Dunn’s multiple comparison tests (*p* < 0.05). Different letters indicate significant differences according to multiple comparison test results. For the reaction at pH 5.5 (**D**; *n* = 3), results were analyzed by the unpaired two-tailed *t*-test (*p* < 0.05). Asterisks indicate significance: ****, *p* < 0.0001. nd = not detected.

All LMW methanolic extracts were able to generate ^•^OH radicals as detected using electron paramagnetic resonance (EPR) in the presence of the DMPO spin-trapping agent. The DMPO-^•^OH adduct displayed its characteristic 4-line spectrum, identified using theoretical simulation (Figure 6A). Note that EPR spectra are typically reported as “relative amplitude” for the Y-axis values. Semi-quantification via spin trapping adduct showed significant differences among *Fmed* strains, with the highest average ^•^OH relative amplitude recorded for strain *Fm6* (7669) and the lowest for *Fm1* (−48.05% compared to *Fm6*) and *Fm2* (−29.04% compared to *Fm6*) (Figure 6C). All other strains generated a statistically comparable relative amount of ^•^OH radical to the *Fm6* strain. The methanolic extracts of *Fmed* strains *Fm1* and *Fm6*, with the least and most ^•^OH produced at pH 3.5, respectively, were also tested for ^•^OH generation at pH 5.5. Both were able to generate ^•^OH, though with lower average relative amplitude compared to pH 3.5 (739 for *Fm1* vs. 2056 for *Fm6*) (Figure 6B,D).

### 3.7. Carbohydrate Oxidative Degradation

**Figure 7:**
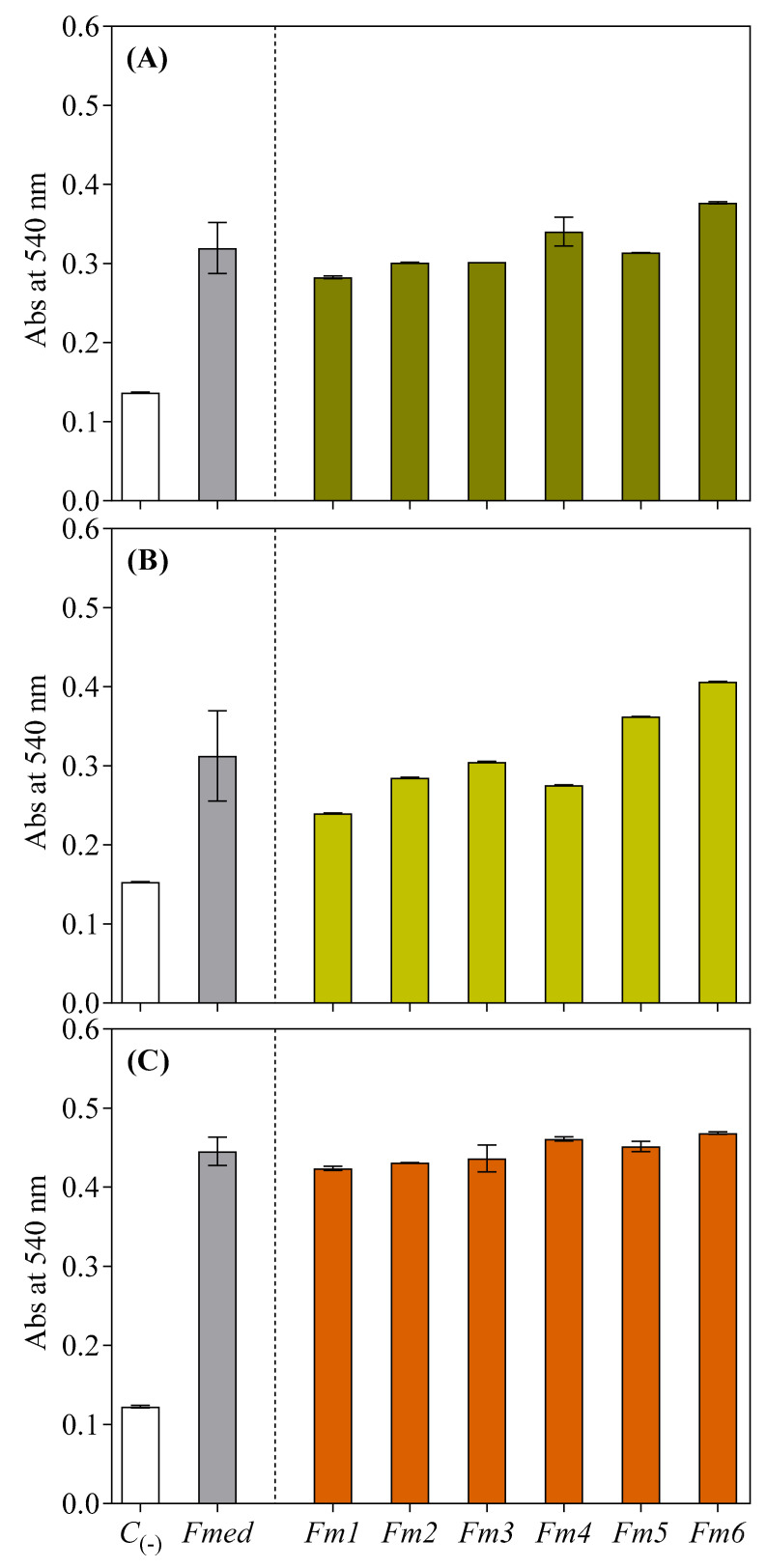
Increase in absorbance (measured spectrophotometrically at 540 nm) from the redox cycling of different LMWCs with (**A**) cellulose, (**B**) hemicellulose, and (**C**) Gewürztraminer wood sawdust. Values for each *Fmed* strain are the means ± standard deviation of 4 pooled replicates. Results from negative controls (*C*_(-)_) and *Fmed* average values are shown on the left.

The results from the experiments on the degradation of hemicellulose (xylan), the cellulose model (microcrystalline cellulose) substrate, and GW-sawdust by extracts of LMWCs from the different fungal strains were associated with an increase in reducing sugar concentration after 12 hrs of the oxidative treatments. All the substrates were more degraded by the redox cycling of the LMW methanolic fraction with respect to controls (containing only ferric iron, H_2_O_2_, and an aliquot of the methanolic extract coming from the mock-inoculated liquid medium). After 12 hrs, average absorbance values (540 nm) of 0.32, 0.31, and 0.44 were initially obtained for cellulose, hemicellulose, and GW-sawdust, respectively (Figure 7). The *Fm6* strain methanolic extract incubated with cellulose produced the highest absorbance value (0.37), while cellulose incubated with the *Fm1* strain methanolic extract had the lowest value (−24.32% compared to *Fm6*). The absorbance of the cellulose mixed with extracts of the other strains fell between the two extremes (Figure 7A). 

For hemicellulose, the highest absorbance value was recorded when incubated with a methanolic extract from the *Fm6* strain (0.38), while the lowest was when incubated with methanolic extracts from strains *Fm4* and *Fm1* (−28.95 and −39.47% compared to *Fm6*, respectively). The absorbance of all other strain extracts was between the two extremes (Figure 7B).

The same trend was recorded for GW-sawdust when incubated with the methanolic extract from strain *Fm6* producing the highest absorbance (0.40), while the lowest was recorded when incubating with the methanolic extract from the strain *Fm1* (−7.5% compared to *Fm6*). Incubation of the GW-sawdust with all other extracts from other strains produced absorbance values that fell between the two extremes (Figure 7C).

## 4. Discussion

*Fomitiporia mediterranea* represents the primary white rot fungus (WRF) in European vineyards affected by the Esca complex of diseases. To the best of our knowledge, this article represents the first description of a non-enzymatic CMF-like pathway adopted by a white rot Basidiomycota species involved in ECD, in line with the current reconsideration of the biomolecular mechanisms in the wood decay binary paradox (white vs. brown rot vs. intermediate species). Our results show how different *Fmed* strains were able to produce LMW phenolic or phenolic-derived compounds that were able to redox cycle with iron when cultivated in vitro in nutrient-restricted and iron-limited conditions often found in wood, and at a pH similar to that found in the wood cell wall. Further, under these conditions, the phenolic-derived compounds were shown to generate H_2_O_2_ and ultimately produce hydroxyl radicals (^•^OH), responsible for lignocellulose constituent oxidation when reacted with holocellulose sources and grapevine wood sawdust. The *Fmed* strain variability was shown to be a factor influencing several stages of the CMF pathway.

When cultivated in a liquid culture designed to reproduce the low nutrient and iron-repressed conditions often found in wood, *Fmed* strains acidified the liquid medium to produce LMWCs, in alignment with previous in vitro observations made by Sparapano et al. [78] and Bruno and Sparapano [57] in static Czapek medium culture. In wood decay induced by Basidiomycota species, the acidification of the microenvironment surrounding fungal hyphae is concomitant with oxalate production as a fundamental step [36,39,45,79,80]. More specifically, for the CMF pathway described for brown rot decay, the low pH and the presence of oxalate are required to chelate and solubilize the unavailable, insoluble Fe(oxydydr)oxide complexes present in the surrounding microenvironment. This promotes the migration of chelated iron into the fungal extracellular matrix. After the transfer of the iron from oxalate to the fungal phenolic chelators, the iron can be reduced, and this occurs within the higher pH wood cell walls where it is required for the subsequent steps of the CMF pathway [29,36,43]. In this study, levels of oxalic acid or other LMW organic acids typically produced by Basidiomycota species during wood decay (i.e., acetate, gluconate, succinate) were not specifically assessed in *Fmed* secretome. However, it is well known that the *Fmed* genome possesses genes coding for enzymes involved in oxalate metabolism regulation, specifically oxalate oxidases and decarboxylases [33].

In the extensive work on non-enzymatic mediated Fenton (CMF) chemistry in lignocellulose degradation, it is well accepted that LMWC-mediated Fe^3+^ reduction to Fe^2+^, and the reaction of the latter species with H_2_O_2_ are critical for hydroxyl radical (^•^OH) generation within the wood cell wall microenvironment to initiate lignocellulose degradation. This subsequently may allow extracellular enzymes to further digest wood cell walls or the oligosaccharides diffusing from those cell walls [29,36,40,41]. However, for WRF, the role of LMWCs has not been clear. Even though some LMWCs have been shown to diffuse into wood cell walls in advance of an enzymatic attack, their role has been mostly considered as intimately-associated mediators for extracellular enzyme action, acting as an electron shuttle or metal chelating cofactor to assist with enzymatic degradation [22,63,81,82,83]. Goodell et al. [84] previously found that many white rot fungi produced iron-reducing compounds when grown on both softwoods and hardwoods, but generally to a lesser extent than classic brown rot fungi. However, the possibility of a LMWC catalytic system in mediated Fenton reactions playing a direct role in white rot or transitional decay species has received far less consideration. In this context, our study represents an advance both in scientific knowledge for wood decay in general and for ECD pathogenesis. 

The LMW methanolic extracts from all *Fmed* liquid cultures tested were able to both chelate and redox cycle with iron, causing a “multiple iron reduction” (MIR) that has previously been reported in other systems [85]. This can function as a catalytic source of H_2_O_2_ in the presence of reduced iron (Fe^2+^), ultimately leading to ^•^OH generation. The levels of MIR attained by the different strains displayed significant differences. 

The total absence of LiP encoding genes in the *Fmed* genome [33] may suggest that a CMF-like pathway involving Fe^3+^-reducing LMWCs could be involved at least partially in lignocellulose degradation found in *Fmed* white rot-affected vines. LiP is one of the Class-II PODs with the highest redox potential, able to oxidize both phenolic and non-phenolic lignin moieties within lignocellulose biomass [29]. Therefore, Fe^3+^-reducing LMWCs could represent a “backup strategy” to compensate for the lack of genes encoding for LiP. Future studies on the interactions of the LMW fungal fractions with enzymatic lignocellulolytic components, together with relative gene expression analysis, are necessary to clarify the roles of the fungal metabolites in grapevine wood undergoing decay in Esca (*sensu strictu*). Apart from the catalytic role of the LMWCs in the CMF pathway of *Fmed*, the possibility that LMWCs may have a mediating role for extracellular enzymes cannot be ignored. In this regard, naturally produced microbial aromatic metabolites mediating laccase activity have been reported for some WRF [29,86]. Concerning H_2_O_2_, our results show an efficient autocatalytic LMWC-mediated molecular oxygen (O_2_) reduction system responsible for H_2_O_2_ generation in the *Fmed* methanolic extracts. This is consistent with systems described for non-enzymatic H_2_O_2_ generation in BRF and, recently, for some Ascomycota species causing soft rot [41,43]. Possible mechanisms explaining a catalytic role for LMW fractions could include (i) the redox cycling of LMWCs with O_2_ to yield ^•^OOH, which can produce H_2_O_2_ via a dismutation reaction [87]; and (ii) the Fe^2+^ produced in the MIR phenomena reducing ^•^OOH generated from O_2_ reduction to yield H_2_O_2_ [88]. Further studies are necessary to elucidate the mechanisms driving H_2_O_2_ generation in *Fmed* methanolic LMW extracts. Another pathway for the generation of H_2_O_2_ has been proposed for wood decay, but it involves enzymes (such as alcohol oxidases or methanol oxidases), which are not able to penetrate the wood cell wall during incipient decay [40,60,89]. Thus, reduction of the O_2_ present in the cell wall via non-enzymatic reactions remains the most efficient path for the generation of H_2_O_2_ in situ, where it is needed to produce ^•^OH. Hydroxyl radicals have a very short half-life (nanoseconds), so they must be generated directly within the cell wall to be active in lignocellulose degradation [29]. Hydroxyl radicals were efficiently generated from the LMW fraction of *Fmed* liquid culture ultrafiltrates, both at pH 5.5 and pH 3.5. These data suggest that ^•^OH generation from LMWC-mediated mechanisms was possible within the intact wood cell wall (where the pH is slightly acidic, around 5.5) and in more acidic conditions, where the decay was more advanced [41]. Our results could potentially explain the diamond-shaped cavities observed by Pacetti et al. [20] and Kassemeyer et al. [58] in the S2 layer of *Fmed*-infested grapevine wood cell walls, as this phenomenon cannot be explained by the activity of enzymes alone in line with an earlier hypothesis of a non-enzymatic pathway for *Fmed* [19]. More studies *in lignum* are necessary for confirmation. 

Ultra-high-performance liquid chromatography coupled with mass spectroscopy analysis allowed the identification of some LMWCs in the ultrafiltered methanolic extracts, including some phenolic acids (hydroxybenzoic and hydroxycinnamic acids), phenolic aldehydes, and other aromatic compounds. The mycotoxin 4-hydroxybenzaldehyde was found previously in the *Fmed* secretome, but no redox cycling ability with iron was attributed to it [54,90]. Other compounds isolated from *Fmed* methanolic extracts were previously found in liquid cultures of BRF, WRF, and soft rot fungi (Ascomycota), including benzoic acid, E4-hydroxycinnamic acid (*p*-coumaric acid), 4-hydroxy-3-methoxybenzoic acid (vanillic acid), 4-hydroxy-3-methoxybenzaldehyde (vanillin), 3-methoxybenzhaldehyde, 2-hydroxybenzoic acid (salicylic acid), and hypholomine B [38,41,91,92]. The involvement of some phenolic molecules, especially catecholate, in driving mediated Fenton reactions is well known [93,94]. For vanillic acid, *p*-coumaric acid, and vanillin, redox cycling with iron has been individually reported [90]. Interestingly, the only compounds possessing catechol moieties identified in our study (the two isomers of hypholomine B) have been described in *Phellinus baumii* as styrylpyrone pigments with iron chelation capacity that also exhibit antioxidant properties [95]. Future experiments assessing Fe^3+^-reducing capacity and pro-oxidant properties will be of great interest as other LMWCs (such as variegatic acid) with both anti- and pro-oxidant properties, depending on pH and transition metal proximity have been reported in the context of CMF reactions induced by the BRF *Serpula lacrimans* [35,39]. Salicylic acid is well known for its antioxidant properties [96], but it has also been reported to function as a pro-oxidant under some conditions [97]. Salicylic acid has recently been used as a radical scavenger to regulate the CMF reaction induced by the LMW fraction secreted by *Eutypa lata (Elata)*, *Phaeoacremonium minimum (Pmin)*, and *Phaeomoniella chlamydospore (Pch)* [42]. In our experiments, ^•^OH production was still evident even when *Fmed* produced salicylic acid, suggesting that it may function as a pro-oxidant under our conditions or that other LMWCs in the system produce more ^•^OH radicals than salicylic acid can scavenge. Other LMWCs (methyl-4-hydroxybenzoate, 5-hydroxyindole-3-acetic acid, and N-acetyl-5-aminosalicylic acid) identified in the *Fmed* methanolic extracts had structures that suggest iron chelation capability. There was also evidence that 5-hydroxyindole-3-acetic acid exhibited both anti- and pro-oxidant properties, and the non-acetylated form of 5-aminosalicylic acid possessed both iron-reducing and pro-oxidant properties, as reported previously [98,99]. However, further experiments with individual model metabolites would be required to demonstrate the redox cycling capacity of these compounds with iron to promote ^•^OH generation. Moreover, several metabolites still need to be identified, and the individual contribution of other catecholates, quinones, and enzyme mediators, such as chlorinated alcohols, cannot be discounted.

Degradation of hemicellulosic (xylan) and cellulosic (microcrystalline cellulose) model substrates was associated with an increase in reducing sugar concentration after oxidative treatments, in accordance with previously reported research by Arantes and Milagres [77] and Arantes et al. [38]. Mediated Fenton reactions with the *Fmed* LMW fraction were faster and more efficient in degrading the substrates than reactions with only iron and hydrogen peroxide. Moreover, our results proved the efficiency of the *Fmed* LMW fraction on the degradation of GW sawdust for the first time. However, while demonstrating the efficiency of the *Fmed* LMW fraction in generating ^•^OH radicals and increasing the quantity of reducing ends of wood carbohydrates (possibly via hydrogen abstraction), these results remain preliminary without providing information about the depolymerization of the substrates. Future experiments are required to understand the kinetics and timeline of holocellulose depolymerization and possible effects on lignin (demethylation/demethoxylation). This information would be of great interest to clarify not only *Fmed*’s decay pattern(s) but also ECD pathogenesis. 

As discussed above, our data represents an advance not only in understanding additional mechanisms associated with a type of white rot decay but also in demystifying “Esca” (*sensu strictu*, currently called “white rot” or “amadou”) pathogenesis, reinforcing the preliminary observations of Di Marco et al. [55] with *Fmed* (formerly classified as *Fomitiporia punctata*) where they describe a positive reaction for the chrome azurol assay (CAS) test in petri dishes. These authors also found iron reduction capacity in their liquid culture supernatant (but without LMW fraction separation) and pioneered the concept that iron (with a subsequent ROS stream) could have a role in “Esca” pathogenesis. Considering the ECD more broadly than just the participation of *Fmed*, other Ascomycota species associated with the complex could potentially also utilize a CMF-like pathway, as demonstrated previously [39], furnishing new insights for understanding typical wood necrosis formation in the ECD. The knowledge gained from our experiments with *Fmed* represents a step forward on the path toward understanding (and potentially controlling) the disease complex. In agreement with the synergistic microbial hypothesis of Bruez et al. [100], it appears that all the major fungi associated with the ECD in the Mediterranean region share a common biochemical pathway. This pathway, namely a CMF-like reaction, may be activated prior to enzymatic action on the lignocellulose biomass to promote the onset of the diseases and consequent hydraulic and mechanical failures. Interfering with ROS generation may potentially serve to control the diseases’ progression, and interestingly, some in vitro trials showed the inhibition of ^•^OH generation when the LMW fractions of *Pch*, *Pmin*, and *Elata* were mixed with antioxidants and chelators, including salicylic acid, EDTA, butylated hydroxy anisole (BHA), and butylated hydroxytoluene (BHT) [42]. 

In general, this study shows that *Fmed* can adopt a non-enzymatic “CMF-like” system to promote pathogenesis and wood necrosis and that significant differences among *Fmed* strains are evident in much of the CMF mechanistic pathway. Indeed, *Fomitiporia mediterranea* is known as a heterothallic species with obligate outcrossing, and because of this, the fungus has remarkable intraspecific variability in vineyards [69]. Interestingly, *Fmed* strain *Fm1*, the only strain originally isolated from a non-*Vitis* host (*Olea europaea* L.), displayed very different behaviour (except for generated H_2_O_2_) compared to the other strains, suggesting a possible degree of host-specificity. Host-specificity has previously been discussed for *Fmed* after cross-pathogenicity tests and the consequent formation of different levels of wood discoloration and necrosis, depending on the host of isolation [101,102]. A deep screening of *Fmed* isolates from non-*Vitis* hosts is necessary to confirm how the host-specificity of different strains may contribute to the CMF pathway. Finally, in our research, the *Fmed* strains produced different suites of LMWCs, with strains *Fm1* and *Fm6* being the least and the most active, respectively, relative to triggering a CMF-like pathway. Specifically, *Fm1* methanolic extracts presented the lowest: (i) capacity for acidification; (ii) production of total phenols; (iii) ferric iron reduction capacity, and (iv) ^•^OH production. The opposite behavior was observed for *Fm6* in all categories. Surprisingly, the levels of H_2_O_2_ generated do not align with the observations for the other data of the CMF pathway. A possible explanation could involve the ratios between individual compounds in the strains’ phenolic profiles. The LMWCs identified did display structures with different multiple coordinating oxygens or different positioning of the hydroxyl groups, suggesting a differential affinity for iron and molecular oxygen in the CMF reaction. Moreover, several compounds in the *Fmed*-methanolic extracts still remain unidentified. It is plausible that some unidentified LMWCs could differentially influence H_2_O_2_ generation, especially if present in different ratios. A similar discrepancy between the results of total phenols, FeRA, and generated hydrogen peroxide has already been observed by Perez-Gonzalez et al. [41] in methanolic extracts from some Ascomycota species. Concerning fungal biomass, at the present stage, it is not possible to formulate a robust hypothesis on its relationship with the CMF pathway for *Fmed*, and research is needed to investigate any potential synergy between primary and secondary metabolism. Differences in growth in an active CMF reaction context may be due to downregulation of primary metabolism when energy is shifted towards phenolic compound biosynthesis, or alternatively, these differences in growth may simply be related to intrinsic nutrient-use efficiency. However, the differences observed at the strain level cannot be assumed to be definitive, as it is known that *Fmed* mycelium can change type during the life span of the fungus, from bleaching (B-Type) to staining (S-Type), with both the physiology and the metabolism of the fungus undergoing change together. It is feasible that such changes could also affect the production of LMW phenolic metabolites in *Fmed*, especially considering that S-Type mycelia induce very strong media pigmentation [15]. 

## 5. Conclusions

Our experiments demonstrate that *Fomitiporia mediterranea*, a WRF responsible for “Esca” *sensu strictu* (a syndrome of the Esca complex of diseases), possesses the means to generate a CMF reaction by secreting an array of LMW-Fe^3+^ reducing compounds. The LMWCs produced were able to: (i) redox cycle with iron; (ii) act as an efficient autocatalytic source for H_2_O_2_ generation; and (iii) generate ^•^OH that can participate in holocellulose oxidation. Intraspecific variation appears to be a factor that impacts several stages of the non-enzymatic CMF pathway. 

We propose that an early-stage non-enzymatic CMF-type degradation of grapevine wood cell walls, promoted by ECD fungi, is required before action by any fungal CAZyme or ligninase enzymes may occur. Non-enzymatic lignocellulose mineralization may continue either with or without enzymatic depolymerization or synergistically with enzyme action (within the fungal extracellular matrix). Simultaneously, methoxylated phenolic compounds derived from the grapevine wood’s degraded lignin may potentially be participating in the previously described multiple iron reduction phenomena, further contributing to ^•^OH generation and lignocellulose degradation [85]. In this context, investigating antioxidant sources for treatment to control ECD in the future could be fruitful.

Future experiments in vitro, in lignum, and in planta are required to clarify whether synergy between non-enzymatic and enzymatic mechanisms occurs in *Fmed*-induced grapevine wood decay and to determine the influence of microenvironmental and abiotic factors on CMF reactions. Finally, the involvement of LMWCs (both secreted by *Fmed* and as a “byproduct of wood degradation”) must be further explored relative to the development of ECD-foliar symptoms.

## Figures and Tables

**Table 1:** Listing of FeRA compounds and annotation levels. Compounds labeled with “†” refer to molecules that have previously been demonstrated to reduce Fe^3+^ to Fe^2+^. Compounds labeled with “/” refer both to unknown and/or described as not able to reduce Fe^3+^ to Fe^2+^. The numbers 1, 2, and 3 in the last column indicate the type of annotation carried out: by chemical standard (1), by the spectral library (2), and with correspondence to chemical formulae in the literature (3).

Compound	FeRA	Type of Annotation
(a) 4-Hydroxybenzaldehyde	/	1
(b) Benzoic acid	/	1
(c) 4-Hydroxy-3-methoxybenzoic acid (vanillic acid)	†	1
(d) 4-Hydroxycinnamic acid (*p*-coumaric acid)	†	1
(e) 2-Hydroxybenzoic acid (salicylic acid)	†	1
(f) 3-Methoxybenzaldehyde	/	1
(g) N-acetyl-5-aminosalicylic acid	/	2
(h) 4-Hydroxy-3-methoxybenzaldehyde (vanillin)	†	2
(i) 5-Hydroxyindole-3-acetic acid	/	2
(j) Methyl-4-hydroxybenzoate	/	2
(k) Hypholomine B (isomers a and b)	/	3

## Data Availability

The data presented in this study are included in the article and the Supplementary Materials. Further inquiries can be addressed to the corresponding authors.

## Supplementary Materials

The following supporting information can be downloaded at: https://www.mdpi.com/article/10.3390/jof9040498/s1, Table S1: List of *Fomitiporia mediterranea* strains used during the experiments, originating from different mycological collections. A key was attributed to each strain to facilitate data interpretation.; Table S2: Statistical analysis of LMW phenol-derived compounds determined by UHPLC-MS/MS. Results were analyzed by ruskal–Wallis test (left-hand superscript “a”) or one-way ANOVA (lefthand superscript “b”) according to data distribution and homoscedasticity, followed by multiple comparison tests (*p* < 0.05), accordingly. Different letters indicate significant differences between *Fmed* strains.

## Appendix A

**Table A1:** In-silico fragmentation of compound (k) annotated as hypholomine B with MetFrag tool [103] embedded in Metaboscape 2021b. 95.7% of the MS/MS spectrum is explained. A comparable MS/MS reference spectrum with the literature was found [104,105].

*m*/*z* Fragment (mDa)	Calculated Formula	Loss	Proposed Structure in Yellow for the Fragment
445.092	C_25_H_17_O_8_^-^	CO_2_	
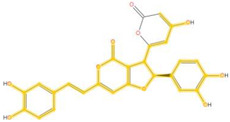
403.082	C_23_H_15_O_7_^-^	C_3_H_2_O_3_	
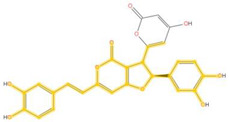
335.055	C_19_H_11_O_6_^-^	C_7_H_6_O_4_	
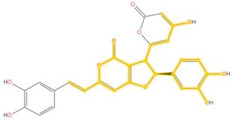
309.040	C_17_H_9_O_6_^-^	C_9_H_8_O_4_	
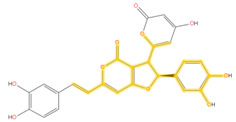
283.062	C_16_H_11_O_5_^-^	C_10_H_6_O_5_	
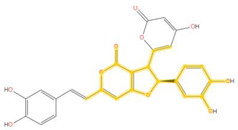
241.050	C_14_H_9_O_4_^-^	C_12_H_8_O_6_	
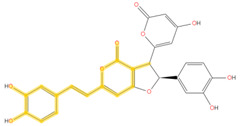
199.040	C_12_H_7_O_3_^-^	C_14_H_10_O_7_	
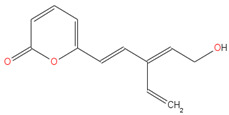
159.045	C_10_H_7_O_2_^-^	C_16_H_10_O_8_	
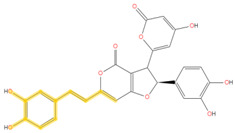
135.045	C_8_H_7_O_2_^-^	C_18_H_10_O_8_	
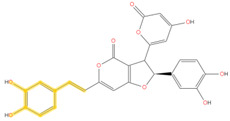

**Table A2:** Average chromatographic retention time (RT), the mass-to-charge ratio (m/z), and mass (M) of the identified compounds in the study.

RT [min]	*m*/*z*	M	Compound Name
8.52	121.029	122.037	(a) 4-Hydroxybenzaldehyde
11.07	121.029	122.037	(b) Benzoic acid
8.58	167.037	168.042	(c) 4-Hydroxy-3-methoxybenzoic acid (vanillic acid)
9.74	163.040	164.047	(d) 4-Hydroxycinnamic acid (*p*-coumaric acid)
11.35	137.024	138.032	(e) 2-Hydroxybenzoic acid (salicylic acid)
9.30	135.045	136.052	(f) 3-Methoxybenzaldehyde
7.54	194.046	195.053	(g) N-Acetyl-5-aminosalicylic acid
9.17	151.040	152.047	(h) 4-Hydroxy-3- methoxybenzaldehyde (vanillin)
9.27	190.051	191.058	(i) 5-Hydroxyindole-3-acetic acid
10.98	151.040	152.047	(j) Methyl-4-hydroxybenzoate
11.80	489.082	490.090	(k) Hypholomine B (isomer a)
12.24	489.083	490.090	(l) Hypholomine B (isomer b)
