# Supplementary material for: First Description of Non-Enzymatic Radical-Generating Mechanisms Adopted by Fomitiporia mediterranea: An Unexplored Pathway of the White Rot Agent of the Esca Complex of Diseases
Source: J Fungi (Basel). 2023 Apr 21;9(4):498. doi: 10.3390/jof9040498 (PMC10143301; doi:10.3390/jof9040498)
Supplement: Supplementary file 1 [file jof-09-00498-s001.zip › Table S1_revised.pdf]

Table S1. List of *Fomitiporia mediterranea* strains used during the experiments, originating from different mycological collections. A key was attributed to each strain, to facilitate data interpretation.

**Key**	**Strain code**	**Isolation locality**	**Collection from which the strain was sourced**	**Host**	**Sampling date**
Fm1	235.03	Riotorto (Livorno), It	Fungal collection PVFi of DAGRI, Section of Plant pathology and entomology (University of Florence, Italy)	*Olea europaea* L.	2003
Fm2	LR71	Vendargues (34), Fr	Fungal collection of lNRAE Montpellier, DAAV team of UMR AGAP (INRAE, Montpellier, France)	*Vitis vinifera* cv Alphonse Lavallée	1996
Fm3	LR2	Beauvoisin (30), Fr	Fungal collection of lNRAE Montpellier, DAAV team of UMR AGAP (INRAE, Montpellier, France)	*Vitis vinifera* cv Carignan	1996
Fm4	LR124	Villeneuve les Maguelone (34), Fr	Fungal collection of lNRAE Montpellier, DAAV team of UMR AGAP (INRAE, Montpellier, France)	*Vitis vinífera* cv Carignan	1996
Fm5	LR85	Ortoffa (66), Fr	Fungal collection of lNRAE Montpellier, DAAV team of UMR AGAP (INRAE, Montpellier, France)	*Vitis vinifera* cv Grenache	1996
Fm6	PHCO36	Saint-Preuil (16), Fr	INRA-UMR 1065 SAVE collection (INRAE, Bordeaux, France)	*Vitis vinifera* cv Ugni blanc	1996
