# Supplementary material for: First Description of Non-Enzymatic Radical-Generating Mechanisms Adopted by Fomitiporia mediterranea: An Unexplored Pathway of the White Rot Agent of the Esca Complex of Diseases
Source: J Fungi (Basel). 2023 Apr 21;9(4):498. doi: 10.3390/jof9040498 (PMC10143301; doi:10.3390/jof9040498)
Supplement: Supplementary file 1 [file jof-09-00498-s001.zip › Table S2_revised.pdf]

Table S2. Statistical analysis of LMW phenol-derived compounds determined by UHPLC-MS/MS. Results were analyzed by Kruskall-Wallis test (left-hand superscript "a") or one-way ANOVA (left-hand superscript "b") according to data distribution and homoscedasticity, followed by multiple comparison tests ($P < 0.05$), accordingly. Different letters indicate significant differences between *Fmed* strains.

**Statistical differences**							
**Compound name**	**P value**	***Fm1***	***Fm2***	***Fm3***	***Fm4***	***Fm5***	***Fm6***
[b] 4-Hydroxybenzaldehyde	< 0.0001	*C*	*B*	*C*	*A*	*C*	*ABC*
[a] Benzoic acid	0.0005	*ABC*	*ABC*	*BC*	*A*	*C*	*AB*
[b] 4-Hydroxy-3-methoxybenzoic acid (vanillic acid)	< 0.0001	*A*	*B*	*B*	*C*	*C*	*A*
[b] 4-Hydroxycinnamic acid (*p*-coumaric acid)	< 0.0001	*C*	*D*	*A*	*E*	*B*	*C*
[a] 2-Hydroxybenzoic acid (salicylic acid)	0.0030	*B*	*AB*	*B*	*A*	*AB*	*AB*
[a] 3-Methoxybenzaldehyde	0.0005	*BC*	*ABC*	*A*	*ABC*	*C*	*AB*
[b] N-Acetyl-5-aminosalicylic acid	<0.0001	*B*	*B*	*B*	*B*	*A*	*AB*
[a] Methyl-4-hydroxybenzoate	0.0010	*A*	*AB*	*B*	*AB*	*B*	*AB*
[a] 4-Hydroxy-3- methoxybenzaldehyde (vanillin)	0.0008	*ABC*	*ABC*	*BC*	*A*	*C*	*AB*
[a] 5-Hydroxyindole-3-acetic acid	0.0006	*A*	*AB*	*BC*	*C*	*ABC*	*ABC*
[a] Hypholomine B (isomer a)	0.0011	*BC*	*ABC*	*A*	*C*	*AB*	*ABC*
[a] Hypholomine B (isomer b)	0.0007	*B*	*AB*	*AB*	*B*	*A*	*AB*
